# Treatment of melanoma with selected inhibitors of signaling kinases effectively reduces proliferation and induces expression of cell cycle inhibitors

**DOI:** 10.1007/s12032-017-1069-0

**Published:** 2017-12-06

**Authors:** Dorota Ciołczyk-Wierzbicka, Dorota Gil, Piotr Laidler

**Affiliations:** Chair of Medical Biochemistry, Jagiellonian University Medical College, ul. Kopernika 7, 31-034 Kraków, Poland

**Keywords:** Melanoma, Proliferation, Cell cycle, Protein kinase inhibitors

## Abstract

Cancer treatment often tends to involve direct targeting enzymes essential for the growth and proliferation of cancer cells. The aim of this study was the recognition of the possible role of selected protein kinases: PI3K, ERK1/2, and mTOR in cell proliferation and cell cycle in malignant melanoma. We investigated the role of protein kinase inhibitors: U0126 (ERK1/2), LY294002 (PI3K), rapamycin (mTOR), everolimus (mTOR), GDC-0879 (B-RAF), and CHIR-99021 (GSK3beta) in cell proliferation and expression of crucial regulatory cell cycle proteins in human melanoma cells: WM793 (VGP) and Lu1205 (metastatic). They were used either individually or in various combinations. The study on the effect of signaling kinases inhibitors on proliferation—BrdU ELISA test after 48–72 h. Their effect on the expression of cell cycle regulatory proteins: cyclin D1 and D3, cyclin-dependent kinase CDK4 and CDK6, and cell cycle inhibitors: p16, p21, and p27, was studied at the protein level (western blot). Treatment of melanoma cells with protein kinase inhibitors led to significantly decreased cell proliferation except the use of a GSK-3β kinase inhibitors—CHIR-99021. The significant decrease in the expression of selected cyclins and cyclin-dependent kinases (CDKs) with parallel increase in the expression of some of cyclin-dependent kinases inhibitors and in consequence meaningful reduction in melanoma cell proliferation by the combinations of inhibitors of signaling kinases clearly showed the crucial role of AKT, ERK 1/2, and mTOR signal transduction in melanoma progression. The results unanimously indicate those pathways as an important target for treatment of melanoma.

## Introduction

Treatment of melanoma causes many problems as melanoma is a heterogeneous disease, resistant to standard chemotherapy, and only subsets of patients respond to systemic therapies [1–3].

The transformation of melanocytes to melanoma cells is characterized by uncontrolled proliferation as a result of abnormalities in cell cycle regulatory mechanisms. In normal cells, the cell cycle is controlled at multiple stages related to DNA replication, cell division, and cell growth [4]. This process also includes mechanisms to ensure that errors are corrected, and if not, the cells commit suicide (apoptosis) [5].

In melanoma, genetic mutations leading to disturbance of these regulatory mechanisms result in uncontrolled cell proliferation [4, 6]. The regulation of cell proliferation is essential for normal development and response to pathological processes such as cell damage and tumorigenesis. Progression through the cell cycle is controlled by cyclins, cyclin-dependent kinases, and inhibitory proteins. Cyclin D1 is usually associated with CDK4, whereas cyclin D3 preferentially partners CDK6 [7].

Cyclins, cyclin-dependent kinases, and inhibitory proteins play an important role in the regulation of cell proliferation, through the G1 restriction point by regulating the function of pRb (retinoblastoma protein) [8].

Cancerous phenotypes result from the dysregulation of more than 500 genes at multiple steps in cell signaling pathways. Most melanomas are driven by BRAF(V600E)-activating mutations [9].

Potential synergy exists between the combination of CDK4/6 inhibitors with existing therapies targeting the MAPK pathway, particularly in subsets of metastatic melanomas such as NRAS and BRAF mutants [3, 10].

In case of V600 BRAF mutation, it seems effective to use RAF inhibitors; RAS and NF1-mutant melanomas have deregulated MEK signaling pathways that are highly sensitive to MEK kinase inhibitors [11], while overexpression of AKT3 isoforms that affects MEK and mTOR signaling pathways has been observed with: wild-type RAS NF1 and Triple Wild-Type cancers, suggesting effective use of target therapy for MEK and PI3K/AKT/mTOR signaling pathway [11].

Activation of mammalian target of rapamycin (mTOR) signaling has been demonstrated in aggressive cancers such as gastric [12] and cervical cancer [13]. The effect of mTOR signaling has also been observed in bladder cancer [14]. The expression of phospho-S6 (a marker of mTOR activity) was found in 55% of muscle-invasive bladder cancers with evident lymph node metastases [15]. mTOR activity was demonstrated to be associated with increased pathological stage and reduced patient survival [15]. Recent research suggests that mTOR mutations often occur in melanoma patients and are of worse therapeutic prognosis [16]. Clinical trials with PI3K/AKT/mTOR pathway inhibitors may be beneficial for melanoma patients with specific mTOR mutations [16].

Understanding melanoma at the molecular level and identifying its novel molecular targets are needed to improve therapeutic strategies. Therefore, the purpose of this study was to recognize the effect of selected signaling kinase inhibitors on melanoma cells proliferation and the expression of cell cycle regulatory proteins.

## Materials and methods

### Cell culture

Human melanoma cell lines: WM793 [vertical-growth phase (VGP)]—Lu1205 (metastatic; biopsy taken from the lung; selection in mice; a culture from the primary site (sternum area) from the same donor as WM793). Cells were cultured in RPMI 1640 medium supplemented with 10% fetal bovine serum and antibiotics: penicillin and streptomycin. Cells were incubated at 37 °C in a humidified atmosphere of 5% CO_2_ in air. Cells were treated with inhibitors: (1) PI3K-LY294002 (Cell Signaling TM)—20 μM concentration, (2) ERK1/2-U0126 (Cell Signaling TM)—10 μM concentration, (3) mTOR—rapamycin (Selleck)—5 nM concentration, (4) mTOR—everolimus (Selleck)—5 nM concentration, (5) B-RAF-GDC-0879 (Selleck)—2 μM concentration, (6) GSK-3β-CHIR-99021(Selleck)—2 μM concentration. Cells were obtained from the ESTDAB Melanoma Cell Bank (Tubingen, Germany).

### Cell proliferation assay

The proliferation of cells was assessed with the BrdU ELISA test (Roche) after 48–72 h, as described previously [17].

### Cytotoxicity assay

Cytotoxicity of PI3K inhibitor—LY294002 (20 μM), ERK1/2 inhibitor—U0126 (10 μM), mTOR inhibitor—rapamycin and everolimus (5 nM), B-RAF-GDC-0879 (2 μM) and GSK-3β-CHIR-99021 (2 μM) assay was determined using Cytotoxicity Detection Kit LDH, Roche, Germany. In all examined melanoma cell lines, inhibitors LY294002, U0126, rapamycin, everolimus, GDC-0879, and CHIR-99021 showed no cytotoxicity effect tested in a culture medium at the time of 72 h. LDH activity in the culture medium in no case exceeded 3%.

### Western blot analysis

Preparation of samples for electrophoresis and western blot analysis as described previously [17]. They used to analyze the following antibodies: Cyclin D1 (#2926 Cell Signaling TM), cyclin D3 (#2936 Cell Signaling TM), CDK4 kinase (#2906 Cell Signaling TM), CDK6 kinase (#3136 Cell Signaling TM), p16 (#4824 Cell Signaling TM), p21(#2946 Cell Signaling TM), p27 (#2552 Cell Signaling TM), and β-actin (A2228, SIGMA).

### Densitometry analysis

Densitometry analyses of western blot analysis were performed on raw volume (sum of intensities of bound—volume calculated from the area of the peak) using SynGene Gene Tools version 4.03.0 (Synoptics Ltd Beacon House, Nuffield Road Cambridge, CB4 1TF, UK).

### Statistics

Cell proliferation data were calculated from mean eight values of three times replicate experiments. All results are presented as experimental mean values which were compared using one-way ANOVA with the Tukey’s post hoc test (Statistica ver. 12, StatSoft); asterisk (*) indicates a significant difference: *p < 0.05, **p < 0.005, ***p < 0.0005.

## Results

### Cell proliferation

The study of the role of protein kinases inhibitors on proliferation of melanoma cells was performed using the BrdU ELISA test after 48–72 h. We did not observe cytotoxic effect of none of the tested protein kinase inhibitors. In the case of each of them used in indicated concentrations: (1) PI3K inhibitor—LY294002 (20 μM), (2) ERK1/2 inhibitor—U0126 (10 μM), (3) mTOR inhibitors—rapamycin and everolimus (both 5 nM), (4) B-RAF inhibitor—GDC-0879 (2 μM), and (5) GSK-3β inhibitor—CHIR-99021 (2 μM) either alone or in various combinations the LDH activity in the media of cells treated for 72 h did not exceed 3%.

**Fig. 1:**
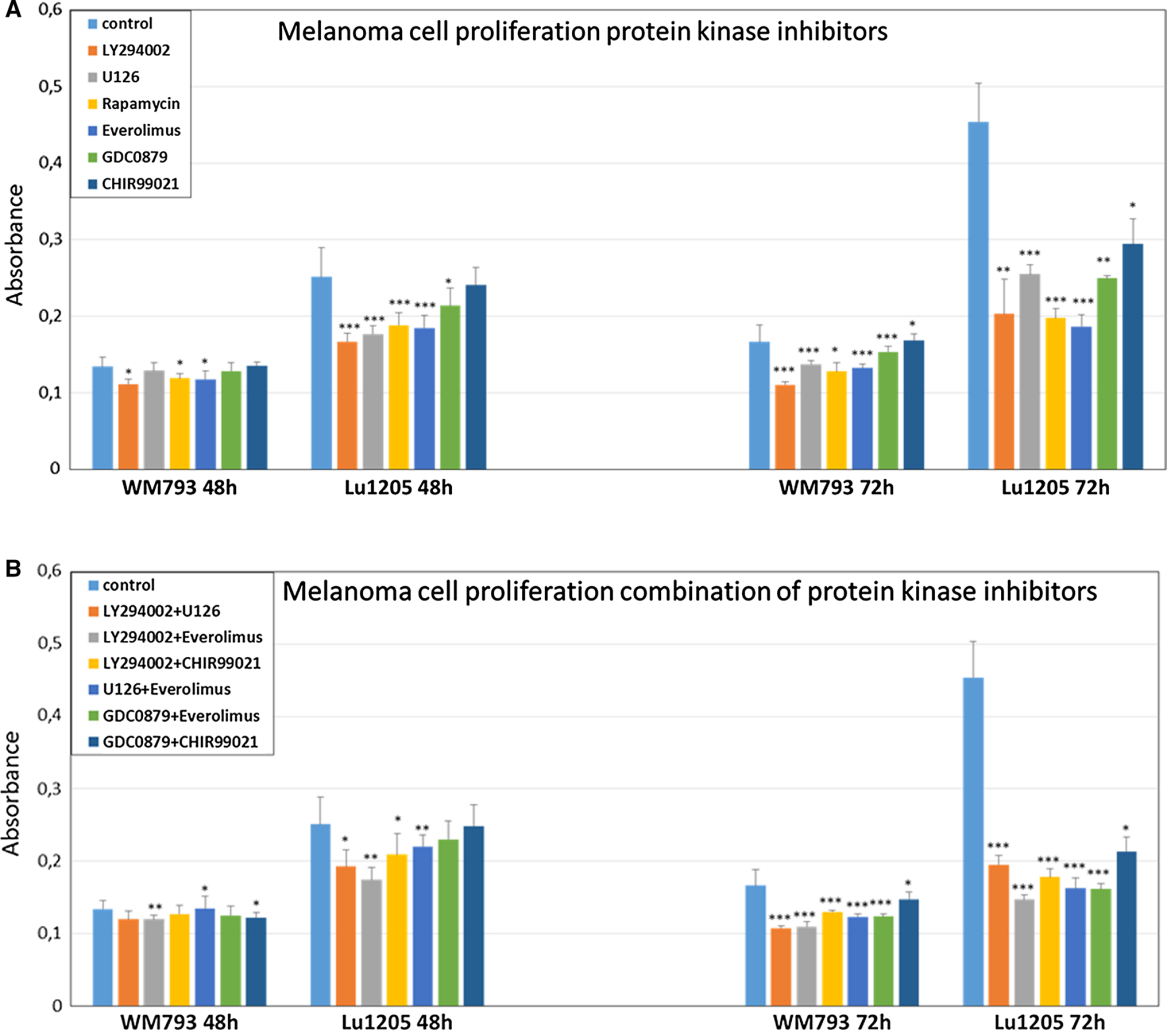
Effect of protein kinase inhibitors on melanoma cell proliferation (**a**). The effect of combination of protein kinase inhibitors on melanoma cells proliferation (**b)**. The proliferation of cells was assessed with the BrdU ELISA test after 48 h and 72 h. Values are expressed as mean ± standard deviation in 8 wells in three independent experiments. All results are presented as experimental mean values which were compared using one-way ANOVA with the Tukey’s post hoc test (Statistica ver. 12, StatSoft); asterisk (*) indicates a significant difference: *p < 0.05, **p < 0.005, ***p < 0.0005

Metastatic melanoma cell line—Lu1205, showed larger decreases in cell proliferation than the primary one, WM793. The treatment of Lu1205 cells with individual inhibitors of mTOR either everolimus (*p* < 0.0005) or rapamycin (*p* < 0.0005) and PI3K inhibitor LY294002 (*p* < 0.0005) for 48 h led to the reduction in their proliferation in the range of 12–34% (Fig. 1a). The use of n GSK-3β kinase inhibitors—CHIR-99021, did not cause any decrease in cell proliferation.

The prolongation of treatment of Lu1205 metastatic melanoma cells up to 72 h led in the case of all the used inhibitors to significant reduction in their proliferation, reaching about 50%, for each of the three of them already mentioned above—everolimus (*p* < 0.0005), rapamycin (*p* < 0.0005), and LY294002 (*p* < 0.005). The treatment of Lu1205 cells with U0126 (ERK1/2 inhibitor) reduced proliferation about 34% (*p* < 0.0005), whereas their treatment with B-RAF inhibitor—GDC-0879, only to about 24% (*p* < 0.005) (Fig. 1a).

The use of the combination of inhibitors U126 (ERK1/2) and GDC-0879 (B-RAF), each with the mTOR inhibitor—everolimus, resulted in more effective, nearly 60% (*p* < 0.0005) reduction in Lu1205 cells proliferation (Fig. 1b). The most profound inhibition was, however, noticed in the case of applications of the combination of mTOR inhibitor—everolimus, with PI3K kinase inhibitor—LY294002, which reduced proliferation of Lu1205 metastatic melanoma cells by 62% (*p* < 0.0005) in (Fig. 1b).

WM793 cells from primary site (VGP) did not respond as effectively as the metastatic ones (Lu1205) to the treatment with each of the inhibitors used in the individual mode since 48-h incubation had almost no effect on their proliferation, while the prolongation of the incubation up to 72 h led to its 25–30% reduction in the case of everolimus (*p* < 0.0005), rapamycin (*p* < 0.05), and LY294002 (*p* < 0.0005) (Fig. 1a).

The use of the various combinations of the studied inhibitors resulted in higher reduction in WM793 cells proliferation, however, not as high as in the case of Lu1205 cells (Fig. 1b). Again in the case of everolimus and LY294002 (*p* < 0.0005) as well as U126 and LY294002 (*p* < 0.0005) used together, the decrease in the proliferation was the highest one, reaching nearly 36% (Fig. 1b).

### Effect of protein kinases inhibitors on cell cycle regulatory proteins in melanoma cells

We also studied the effect of using the individual inhibitors: PI3K inhibitor—LY294002, ERK1/2-U0126; mTOR: rapamycin and everolimus; B-RAF-GDC-0879 and GSK-3β-CHIR-99021; and their combination on the expression of cell cycle regulatory proteins: cyclin D1 and D3, cyclin-dependent kinase: CDK4, CDK6, and cell cycle inhibitors: p16, p21, and p27.

**Fig. 2:**
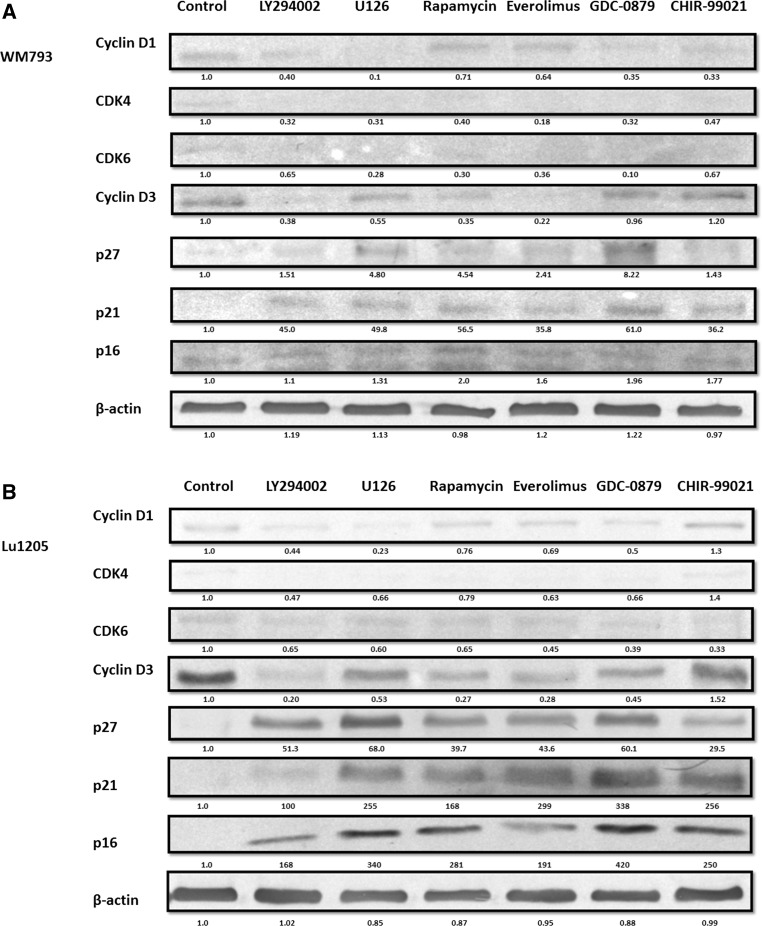
Effect of protein kinase inhibitors on cell cycle protein in melanoma cells. Melanoma cell lines: **a** WM793, **b** Lu1205 were treated with protein kinase inhibitors. Cell cycle protein expression was analyzed by western blot. Densitometry analyses of western blot were performed on raw volume (sum of intensities of bound-volume calculated from the area of the peak) using SynGene Gene Tools version 4.03.0 (Synoptics Ltd Beacon House, Nuffield Road Cambridge, CB4 1TF, UK). Densitometry was used to normalize to control (melanoma cells untreated with protein kinase inhibitors). Presented are representative of at least three independent experiments with similar results

In both studied melanoma cell lines expression of cyclins D1 and D3, cyclin-dependent kinases CDK4 and CDK6 were observed (Fig. 2a, b). The level of the expression of cyclin D3 in untreated cells was much higher than the level of cyclin D1, and it was much higher for both cyclins: D1 and D3 in the case of metastatic Lu 1205 cells than in WM793 primary ones.

The level of cyclin D3 and cyclin-dependent kinase 6 (CDK6) was most decreased after the application of mTOR inhibitors: rapamycin or everolimus (~ 60–75% relative to the untreated cells). Similar results were obtained after application of inhibitor LY294002 (~ 60–70% reduction). CHIR-99021-GSK-3β inhibitor did not affect the level of kinase CDK6 and cyclin D3 (Fig. 2a, b).

In the case of the use of single protein kinase inhibitors, largest decreases in cyclin D1 and cyclin-dependent kinase 4 (CDK4) expression were observed for ERK1/2-U126 and B-RAF-GDC-0879—about 50–70% relative to the control; a little smaller effect was observed for PI3K kinase inhibitor—LY294002—about 50%, and for the inhibitor of GSK-3β kinase—CHIR-99021, the effect was not observed (Fig. 2a, b).

The expression of the cell cycle inhibitors p16, p21, and p27 in untreated (control) cells was initially low in both studied melanoma cell lines. Their level increased upon application of each of the tested protein kinases inhibitors, yet the greatest increase was observed following the use of mTOR inhibitors: rapamycin and everolimus—about 70%, and B-RAF inhibitor GDC-0879—about 60% (Fig. 2a, b).

**Fig. 3:**
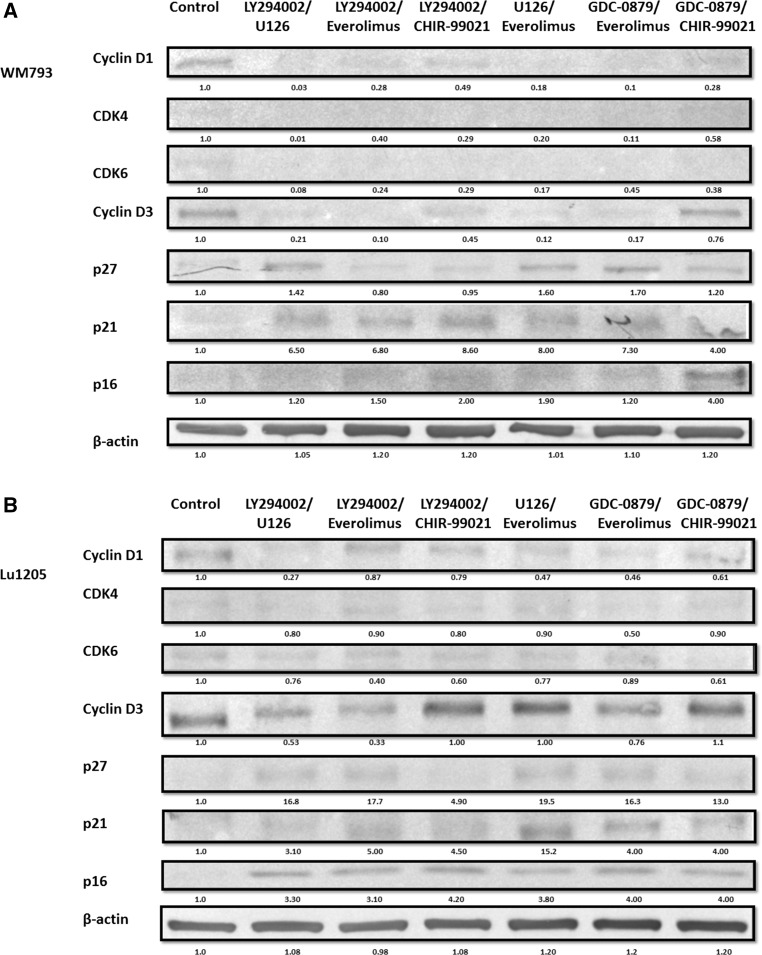
Effect of combination of protein kinase inhibitors on cell cycle protein in melanoma cells. Melanoma cell lines: **a** WM793, **b** Lu1205 were treated with combination of protein kinase inhibitors. Cell cycle protein expression was analyzed by western blot. Densitometry analyses of western blot were performed on raw volume (sum of intensities of bound-volume calculated from the area of the peak) using SynGene Gene Tools version 4.03.0 (Synoptics Ltd Beacon House, Nuffield Road Cambridge, CB4 1TF, UK). Densitometry was used to normalize to control (melanoma cells untreated with protein kinase inhibitors). Presented are representative of at least three independent experiments with similar results

The greatest simultaneous decline of cyclin D1, CDK4 kinase, and cyclin D3 and CDK6 kinase was observed after treatment of melanoma cell with the combination of inhibitors: mTOR—everolimus, with ERK1/2 inhibitor—U126 or B-RAF-GDC-0879 (Fig. 3a, b). The use of the combination of inhibitors everolimus and LY294002 gave noticeable effects on decrease in the level of cyclin D3 and CDK6 kinase by 60–80%, while the use of the combination of inhibitors U126 and LY294002 caused a similar decline in cyclin D1 and CDK4 kinase (Fig. 3a, b).

The decline in the level of cyclins D1 and D3, and cyclin-dependent kinases (CDK4, CDK6) was accompanied by the increase in the level of protein: p16, p21, and p27 (Fig. 3a, b).

## Discussion

In this study, we were able to show that all but one selected (tested) protein kinases inhibitors: PI3K (LY294002), ERK1/2 (U0126), mTOR (rapamycin and everolimus), B-RAF (GDC-0879), and GSK-3β (CHIR-99021) used individually or in combination had strong anti-proliferative effect on melanoma cell lines. In the case of treatment of melanoma cells with each of inhibitors used in individual mode, the best results were obtained for mTOR inhibitor (either everolimus or rapamycin). It is worth to notice that the significant reduction in proliferation was observed for as low as 5 nM inhibitor’s concentration which lays within the range of recommended dose for treatment of human ovarian cancer (3–8 nM) [18].

A little less effective in that respect were PI3K and ERK1/2 inhibitors—LY294002 and U126, respectively.

In the case of an inhibitor of GSK-3β (CHIR-99021), no effect on proliferation after 48-h treatment was found. Similar results on the use of CHIR-99021 inhibitor were reported by [19]; GSK-3β inhibition promotes proliferation and neuronal differentiation of human-induced pluripotent stem cell-derived neural progenitors [20]. On the other hand, induction of apoptosis in pancreatic cancer cells was observed after application of GSK-3β (CHIR-99021) kinase inhibitor [21].

We therefore sought to see if inhibition of that important kinase might have any effect in the case of melanoma cells.

Application of the combination of protein kinase inhibitors gave better results than the use of inhibitors individually. The best results were obtained while using a combination of everolimus and LY294002. Similar results were also obtained for the combination of everolimus and U126 or GDC-0879.

The use of signaling kinases inhibitors were also shown to effect the expression of proteins that are involved in the regulation of the cell cycle which justifies their influence on melanoma cells proliferation. Treatment of melanoma cells with inhibitor of ERK1/2 kinase—U126 and B-RAF-GDC-0879, resulted in the most profound reduction in expression of cyclin D1 and CDK4 kinase but only slightly affected the level of cyclin D3 and CDK6 kinase. The use of mTOR inhibitors everolimus or rapamycin led to the opposite effect and resulted in a significant decrease in the level of cyclin D3 and CDK6 kinase while to only moderate decrease in cyclin D1 and CDK4 kinase levels.

Those observations remain in concert with recently reported results of preclinical studies carried out by a number of centers with the use of ERK1/2 inhibitors which confirm that the decrease in the forms of phosphorylated ERK-pERK is accompanied by the decrease in the expression of cyclin D1 [22, 23]. The role of cyclin D1 in melanoma cells is, however, controversial. In many studies, expression of cyclin D1 is enhanced in primary and metastatic melanoma [7, 24]. On contrary, some histopathological data do not show a statistically significant difference in the level of this cyclin [25], and others suggest that cyclin D1 expression significantly increases due to tumor progression yet decreases in metastases [26]. Some authors suggest that expression of cyclin D1 is the effect of upregulation by constitutive B-RAF-MEK-ERK1/2 signaling initiated by mutant B-RAF [7, 26, 27]. Such relation between hyperactive signaling pathway and cyclin D1 expression may explain its relatively low sensitivity to PI3K and mTOR inhibitors treatment.

According to the literature, the level of cyclin D3 is significantly higher in melanoma than in dysplastic nevi. It can therefore be used as a diagnostic marker for differentiation of these lesions in histologically doubtful cases [8]. Flørenes et al. [28] have shown that expression of cyclin D3 is an important factor in predicting the clinical outcome for patients with superficial spreading melanoma, whereas the level of cyclin D1 expression has no impact on tumor progression. The expression of cyclin D3 in melanoma cells is associated with regulation of the cell cycle at the G1-S phase, which is necessary for efficient entry into S phase, increased cell proliferation, and is a poor prognostic factor [7, 8, 28]. The data obtained by Spofford et al. [7] suggest that the activity of cyclin D3 is regulated by fibronectin-mediated PI3K signaling but not ERK1/2 one, which may explain relatively small decrease in the level of cyclin D3 after the application of U126.

The use of protein kinase inhibitors decreased the level of cyclin D1 and D3 and the cyclin-dependent kinase CDK4 and CDK6, which was accompanied by increased expression of cell cycle inhibitors p16, p21, and p27.

The highest increase in p16 suppressor protein was observed using U126-ERK1/2 inhibitor and rapamycin or everolimus—mTOR inhibitors, a significant increase was also observed after application B-RAF inhibitor—GDC-0879.

Monahan et al. [29] observed potent cooperation between the loss of somatic p16 and acceleration of melanoma genesis, a finding consistent with the view of more prominent tumor suppressor role of p16 relative to p53 in human melanoma. Loss of p16-Rb and ARF-p53 tumor suppressor pathways, as well as activation of RAS–RAF signaling, is seen in a majority of human melanomas [29].

Use of the combination of inhibitors of signaling kinases led to a greater decrease in the level of cyclin D1 and D3 and the cyclin-dependent kinase CDK4 and CDK6 than the application of inhibitors in single mode. The largest decreases in the level of both cyclin D1 and D3 and proteins CDK4 and CDK6 were observed after treatment with the following combination of inhibitors: everolimus and B-RAF inhibitor GDC-0879 or everolimus with ERK1/2 inhibitor U126. Simultaneous application of inhibitor everolimus and that of PI3K-LY29004 gave limited decrease in expression of cyclin D1 and CDK4 kinase but significantly decreased the level of cyclin D3 and CDK6 kinase. However, using a combination of inhibitors of PI3K-LY29004 and ERK1/2-U126 resulted in decreased expression of cyclin D1 and CDK4 and slight decrease in the level of cyclin D3 and CDK6 kinase. The decline in the level of cyclin is accompanied by an increase in the level of tumor suppressor proteins p16, p21, and p27. The highest increase tumor suppressor protein was observed in combinations: mTOR inhibitor—everolimus and ERK1/2-U126, slightly lower for the everolimus and GDC-0879.

Everolimus is a new hope for patients with breast cancer [30]. mTOR inhibitor everolimus (RAD001), an orally administered drug, was recently approved by the US-FDA in combination with exemestane (aromatase inhibitors) for treatment of “hormone receptor-positive” (HR-positive) metastatic breast cancer [31, 32]. Study conducted by a group of Lui et al. [33] demonstrated that everolimus inhibits the proliferation of aromatase inhibitor-resistant breast cancer cells through the downregulation of estrogen receptor expression. Another study [15] showed that everolimus exerts anticancer activity in bladder cancer cells and proposes its role in effective disturbing the growth of bladder cancer cells and T cell lymphoma [34]. Research carried out of animal models have demonstrated that agents targeting mTOR pathway can lead to significant inhibition of proliferation, differentiation, and tumor progression in specific pancreatic ductal adenocarcinoma subpopulations [35].

The presented study is up to our knowledge the only such extensive an promising attempt to recognize and determine which inhibitors of most important signaling kinases as well as their combinations could be in future effectively used in treatment of various subtypes of cutaneous melanoma.
